# Investigation on the Cyclic Response of Superelastic Shape Memory Alloy (SMA) Slit Damper Devices Simulated by Quasi-Static Finite Element (FE) Analyses

**DOI:** 10.3390/ma7021122

**Published:** 2014-02-11

**Authors:** Jong Wan Hu

**Affiliations:** 1 Department of Civil and Environmental Engineering, Incheon National University, 12-1 Songdo-dong, Yeonsu-gu, Incheon 406-840, Korea; 2 Incheon Disaster Prevention Research Center, Incheon National University, 12-1 Songdo-dong, Yeonsu-gu, Incheon 406-840, Korea; E-Mail: jongp24@incheon.ac.kr

**Keywords:** slit damper, superelastic effect, shape memory alloy (SMA), finite element (FE), recentering behavior

## Abstract

In this paper, the superelastic shape memory alloy (SMA) slit damper system as an alternative design approach for steel structures is intended to be evaluated with respect to inelastic behavior simulated by refined finite element (FE) analyses. Although the steel slit dampers conventionally used for aseismic design are able to dissipate a considerable amount of energy generated by the plastic yielding of the base materials, large permanent deformation may occur in the entire structure. After strong seismic events, extra damage repair costs are required to restore the original configuration and to replace defective devices with new ones. Innovative slit dampers fabricated by superelastic SMAs that automatically recover their initial conditions only by the removal of stresses without heat treatment are introduced with a view toward mitigating the problem of permanent deformation. The cyclically tested FE models are calibrated to experimental results for the purpose of predicting accurate behavior. This study also focuses on the material constitutive model that is able to reproduce the inherent behavior of superelastic SMA materials by taking phase transformation between austenite and martensite into consideration. The responses of SMA slit dampers are compared to those of steel slit dampers. Axial stress and strain components are also investigated on the FE models under cyclic loading in an effort to validate the adequacy of FE modeling and then to compare between two slit damper systems. It can be shown that SMA slit dampers exhibit many structural advantages in terms of ultimate strength, moderate energy dissipation and recentering capability.

## 1. Introduction

Steel slit dampers that can be integrated with general seismic resistant systems, such as special and ordinary moment-resisting frames, have been utilized as easily replaceable energy dissipation devices with the intention of protecting the main structural members (e.g., beams and columns) [1–4]. Inelastic deformations in the main structural members make it difficult to repair seismic damage and, hence, require the rebuilding of the building structure [5,6]. Therefore, these devices that dissipate energy based on the yielding of standard base steel sections are designed to concentrate significant inelastic deformations under severe earthquake events. Such a design methodology takes advantage of acceptable seismic performance with respect to economy and safety [7,8]. In spite of the damage control obtained by energy dissipation devices (*i.e.*, steel slit dampers), their permanent deformations still give rise to residual inter-story drifts in the whole moment-resisting frame. Conventional passive control systems with steel energy dissipation devices cannot adequately supply the demand for harmonization between structural and non-structural damage, and thus, adding strength and stiffness to the frame structure shall be required for aseismic design in order to reduce story drifts. Some scientists emphasize that non-structural damage related to residual inter-story drifts is more dangerous than damage related to structural member failure [9,10]. In particular, a recent report study highlights that if the frame system undergoes a residual inter-story drift greater than 0.5%, the owners of buildings in Japan had better rebuild the whole structure from an economic point of view rather than repair them [9]. For this motivation, this study mainly focuses on the slit damper device with recentering capability, so as to considerably decrease permanent deformation in the steel frame structure.

**Figure 1.**
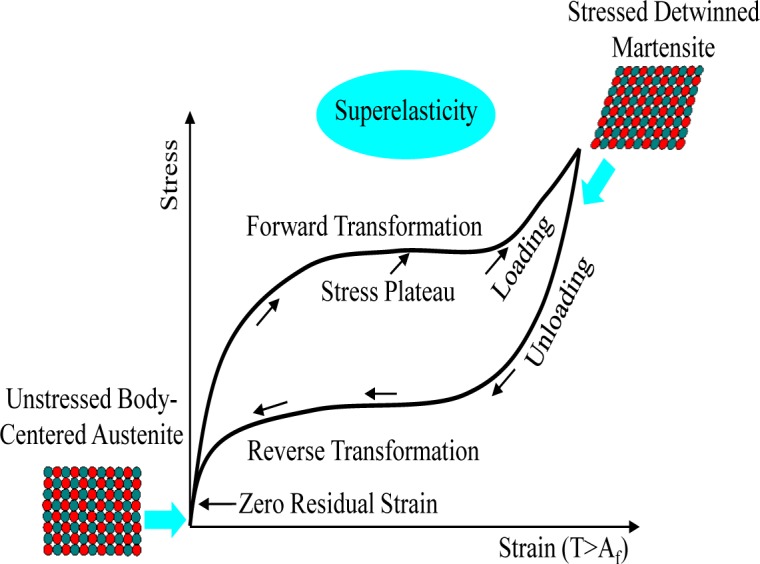
Stress and strain curve for superelastic shape memory alloy (SMA) materials.

One of the best ways to improve seismic performance as regards vibration control and the self-centering effect can be achieved by the utilization of smart materials in aseismic design. Superelastic shape memory alloys (SMAs) have currently been prevalent as smart materials used for seismic control devices in that they exhibit unique material behavior characterized by a flag-shape hysteresis under cyclic loading. The hysteretic behavior of superelastic SMA materials is illustrated in Figure 1. The general SMA composed of a metallic alloy of nickel and titanium, which is referred to as Nitinol, shows superelasticity (or pseudo-elasticity) that is able to recover the original shape only by the removal of stress upon unloading. As shown in the figure, superelastic Nitinol SMAs that typically occur at a temperature limit above the austenite phase transformation (A_f_) do not exhibit any residual deformation without additional heating, even after applying substantial strain, ranging from 6% to 8%. This material behavior makes a significant contribution toward providing an excellent recentering capability, as well as supplemental energy dissipation for the entire frame structure, when such superelastic SMA materials are used in the damper device [11,12]. In this study, slit damper devices fabricated with superelastic SMA materials are consequently introduced to attain both the establishment of additional damping and the mitigation of residual inter-story drifts. The behavior of superelastic SMA slit dampers are compared with that of conventional steel slit dampers after performing finite element (FE) analyses. In addition to the user-material (UMAT) model for reproducing the material behavior of superelastic SMA materials, FE models are additionally calibrated to experimental results with the aim of obtaining a reliable prediction. Finally, both types of slit damper devices, which are compared to each other, are simultaneously evaluated for ultimate strength and recentering capability in order to verify SMA’s superior effect.

## 2. Sample Slit Damper Specimens

**Figure 2.**
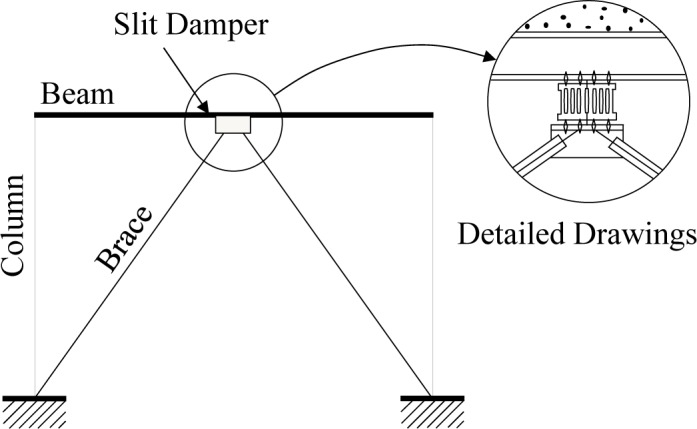
Installation of the slit damper at the concentrically braced frame structure.

**Figure 3.**
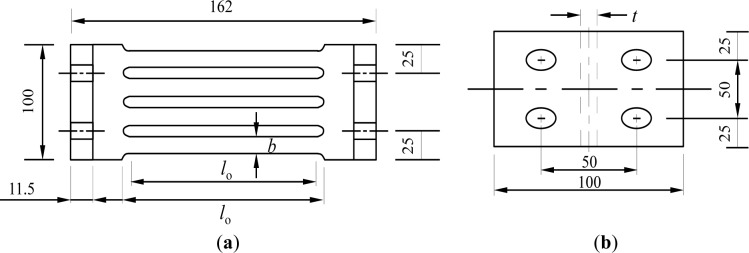
Geometric details for experimental slit damper models: (**a**) front view and (**b**) plan view.

Typical slit damper devices can be installed on top of an inverted-V brace at the concentrically-braced frame structure and connected to the middle of the beam member, as shown in Figure 2. Detailed drawings of the slit damper devices are also presented in Figure 3. They are manufactured from the short length of standard I-shape sections, with a number of slits cut from the web and leaving strips between two flanges. The strips are fabricated to be circular at their ends for the purpose of mitigating stress concentration at the corners. The flange of the slit damper device, where four bolt holes are drilled, is attached to the frame by using weld-free bolts and nuts, thereby eliminating failure uncertainties due to welding [1,3].

**Figure 4.**
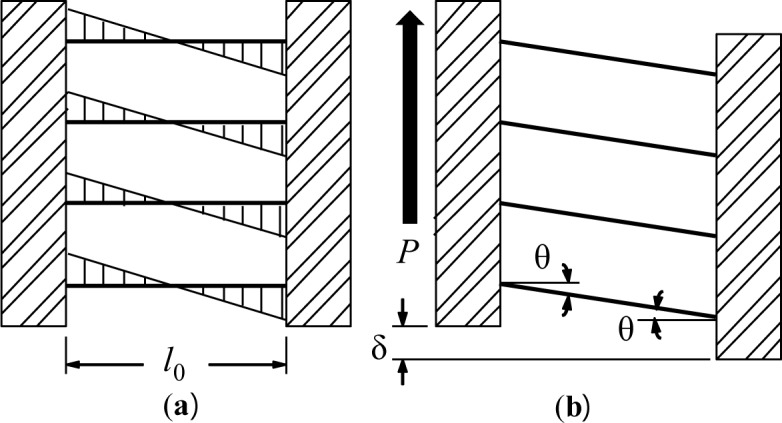
Response mechanism of the slit damper model: (**a**) bending moment diagram and (**b**) deformation shape.

This device directly copes with shear forces transferred from the frame members (*P*) and the corresponding deformation (δ). The strips behave as fixed-end beams under relatively large displacement between two supported flanges. The bending mechanism of the strips is shown in Figure 4. Plastic hinges are likely to form at both ends of individual strips subjected to sufficient displacement. Thus, a significant amount of energy can be dissipated owing to these plastic hinges under the bending mechanism. The required parameters to describe the mechanical response of the slit damper, *i.e.*, strip length (*l*_0_); strip depth (*b*) and web thickness (*t*), are also presented in Figure 3. The yield load of the slit damper (*P*_y_) can be defined under the plastic bending mechanism with the assumption of perfectly elasto-plastic material behavior as follows:

$$M_{P}=\sigma_{y}\frac{tb^{2}}{4}$$ (1)

$$P_{y}=\frac{2nM_{P}}{l_{0}}=\frac{n\sigma_{y}tb^{2}}{2l_{0}}$$ (2)

where *M*_P_ indicates the full plastic moment when plastic hinges form at both ends of each strip with a rotation of θ_p_ and *n* indicates the number of strips in the damper device. The stiffness of the slit damper device can be defined on the basis of an assumption that individual strips are fully constrained at their ends. It is determined as follows:

$$K=n\frac{12EI}{l_{0}^{3}}=n\frac{Etb^{3}}{l_{0}^{3}}$$ (3)

where *I* is the moment inertia of the prismatic strip.

The experimental tests related to the slit damper devices were conducted by Chan and Albermani [3] with the intention of examining not only cyclic responses, but also the structural characteristics, and then, the effects of geometric design parameters were also investigated to identify changes in stiffness and strength. The FE models used for simulating the behavior of the slit damper devices are calibrated to these experimental test results, so as to verify the adequacy of the modeling. A summary of the experimental specimens is given to Table 1. The design parameters, defined as the measured dimensions in the table, are similar to the ones presented in Figure 3. In this paper, six specimens out of a total of nine specimens are selected for the calibration and parametric study. All presented specimens were fabricated, cut from a standard steel wide-flange section, *i.e.*, 161.8 mm (depth) × 152.2 mm (flange width) × 8 mm (web thickness) × 11.5 mm (flange thickness) [3]. The standard coupons used to determine the material properties were obtained from the web. After coupon tests, the average yield stress and average elastic modulus were taken as 316.5 MPa and 206.1 GPa, respectively. As presented in the table, the specimens are classified according to varied *b*/*l*_0_ ratios ranging from 0.155 to 0.215. The original specimens made by Gr. (Grade) 50 carbon steel are labeled from SL1 (SL: Slit Damper) to SL6. On the other hand, the proposed specimens fabricated with superelastic SMA materials are additionally labeled as “-SMA” in the last acronym of the model identification (ID).

## 3. Finite Element Models

**Figure 5.**
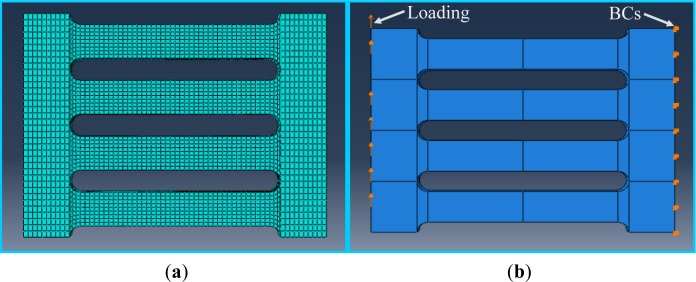
Three-dimensional finite element (FE) models for nonlinear analyses. BC, boundary condition. (**a**) Element mesh and (**b**) loading and BCs.

The ABAQUS (Nonlinear FE Code Program) [13] was used to predict the cyclic response of slit damper devices. FE models were made up of 3D solid elements (*i.e.*, C3D8: 3-dimensional 8-node linear brick element in the ABAQUS program) incorporating fully nonlinear material properties, geometric nonlinearity and displacement-controlled loading. Figure 5 shows 3D FE models concerning element mesh, displacement loading and boundary conditions (BCs). The structural meshes generated by dividing the part were used to make a uniform element size in the FE model. The flange of the slip damper was assumed to be rigid, and accordingly, detailed modeling for a supported flange was replaced with the BCs. Displacement loading was directly imposed on the end of the web, as well, instead of flange modeling. The history of cyclic displacement loading for quasi-static FE analyses was simulated using the static step and the default amplitude function associated with BCs in the program. For each specimen, FE analyses were carried out with similar loading histories to the experimental tests.

The elasto-plastic material behavior with the combination of isotropic and kinematic strain hardening was assigned to FE models for steel slit damper devices. The nonlinear isotropic/kinematic hardening material model, which includes some physical features, such as the Bauschinger effect, plastic shakedown, ratcheting and stress relaxation [13], was selected to simulate the behavior of steel materials in the cyclic loading condition. On the other hand, to simulate the cyclic behavior of superelastic SMA materials, the user material (UMAT) subroutine based on Aurrichio’s model [14,15] was employed in the absence of adequate built-in material models provided by the program. Aurrichio’s material model reflects forward and reverse phase transformation involved in superelasticity under isothermal conditions. It was also based on the concept of generalized plasticity [16–18].

## 4. UMAT Equations and Simulation

In the UMAT subroutine, the degree of phase transformation was represented by an internal variable that may track the fraction of martensite distribution. The internal variables also include transformation strain and equivalent stress-strain relation. Two phase transformation processes, which are divided according to the martensite fraction (ν*_S_*) ranging from zero to one, are necessary to define: (1) transformation from austenite to martensite (*A* → *S*); and (2) transformation from martensite to austenite (*S* → *A*). The linear kinetic rules with respect to the uniaxial stress (σ) are applied to forward transformation (*A* → *S*) as follows:

$$\sigma_{S}^{AS}<\left|\sigma \right|<\sigma_{f}^{AS}\;\text{and}\;\bar{\left|\sigma \right|}>0$$ (4)

where $\sigma_{s}^{AS}$ indicates martensite start stress; $\sigma_{f}^{AS}$ indicates martensite finish stress; | | represents an absolute value and a superposed dot denotes a time derivative. The corresponding time continuous evolution equation can be defined by the relation, as follows:

$${\dot{\nu }}_{s}=-(1-\nu_{s})\frac{\bar{\left|\sigma \right|}}{\left|\sigma \right|-\sigma_{f}^{AS}}$$ (5)

The condition for reverse transformation (S → A) and the corresponding evolution equation can be defined as follows:

$$\sigma_{f}^{\text{SA}}<\left|\sigma \right|<\sigma_{f}^{\text{SA}}\;\text{and}\;\bar{\left|\sigma \right|}<0$$ (6)

$${\dot{\nu }}_{s}=\nu_{s}\frac{\bar{\left|\sigma \right|}}{\left|\sigma \right|-\sigma_{f}^{\text{SA}}}$$ (7)

where $\sigma_{f}^{SA}$ indicates austenite start stress and $\sigma_{s}^{SA}$ indicates austenite finish stress. Total strain can be decomposed into two components, (a) a purely linear-elastic component (ε*^e^*) and (b) a transformation strain component (ε*_L_*) as follows:

$$\varepsilon =\varepsilon^{e}+\varepsilon_{L}\nu_{S}\;sgn(\varepsilon )$$ (8)

Where *sgn*() is the sign function. As shown in Equation (8), the amount of plastic strain is proportional to the martensite fraction. Total strain is assumed to be a control variable. The elastic stress is linearly related to elastic strain. The constitutive equation is written with the elastic modulus (*E*).

$$\sigma =Ee^{e}$$ (9)

The increment of the martensite fraction within discrete time (λ*_S_*) is obtained by integrating the ration of the fraction as follows:

$$\nu_{S}=\nu_{S,n}+\lambda_{S}\;\text{or}\;\lambda_{S}=\int_{t_{n}}^{t_{n+1}}{\dot{\nu }}_{S}dt$$ (10)

where the subscript, *n*, denotes a quantity estimated at time (*t*) and *t_n_*_+1_ is the time value of interest immediately after *t_n_*. Equation (9) can be rewritten based on the linearization of the strain components as follows:

$$\sigma =E\left[\varepsilon -\varepsilon_{L}\;sgn(\varepsilon )\nu_{S}\right]$$ (11)

$$d\sigma =E\left[d\varepsilon -\varepsilon_{L}sgn(\varepsilon )d\lambda_{S}\right]$$ (12)

The quantity of λ*_S_* is proportional to that of plastic strain after yielding, thereby defining:

$$d\lambda_{S}=Hd\varepsilon$$ (13)

where *H* indicates the scalar quantity for the tangent modulus after yielding. Using this relation, between plastic strain and martensite fraction increment, Equation (12) can be converted as follows:

$$d\sigma =E^{T}d\varepsilon$$ (14)

The tangent modulus (*E^T^*) can be rewritten as below.

$$E^{T}=E[1-\varepsilon_{L}H\;sgn\text{(}\varepsilon \text{)}]$$ (15)

The scalar quantity (*H*) used to evaluate the tangent modulus can be computed using the linearization of evolution equations consistent with phase transformation (Equations (8) and (10)) and defined as follows:

$$H=H^{AS}=\frac{-sgn\text{(}\varepsilon \text{)}\left(1-\nu_{S,n}\right)E}{\left(1-\nu_{S,n}\right)[-sgn\text{(}\varepsilon \text{)}E\varepsilon_{L}]+\sigma_{n}-sgn\text{(}\varepsilon \text{)}\sigma_{f}^{AS}}$$ (16)

$$H=H^{SA}=\frac{sgn(\varepsilon )\nu_{S,n}E}{\nu_{S,n}[sgn(\varepsilon )E\varepsilon_{L}]+\sigma_{n}-sgn(\varepsilon )\sigma_{f}^{SA}}$$ (17)

Using these time-discrete evolutionary equations, martensite fractions during each phase transformation process are obtained as follows:

$$\nu =\nu_{S}^{AS}=\frac{\nu_{S,n}E\varepsilon -sgn(\varepsilon )\nu_{S,n}\sigma_{f}^{AS}-E\varepsilon +\sigma_{n}}{-sgn(\varepsilon )\sigma_{f}^{AS}+sgn(\varepsilon )\nu_{S,n}E\varepsilon_{L}-sgn(\varepsilon )E\varepsilon_{L}+\sigma_{n}}$$ (18)

$$\nu =\nu_{S}^{SA}=\frac{\nu_{S,n}E\varepsilon -sgn{\text{(ε)ν}}_{S,n}\sigma_{f}^{SA}}{-sgn{\text{(ε)σ}}_{f}^{AS}+sgn{\text{(ε)ν}}_{S,n}E\varepsilon_{L}+\sigma_{n}}$$ (19)

Finally, the critical strains at the start of martensite, the finish of martensite, the start of austenite and the finish of austenite are determined as follows:

$$\varepsilon_{S}^{AS}=sgn(\varepsilon )\frac{\sigma_{S}^{AS}}{E}+sgn(\varepsilon )\nu_{S,n}\varepsilon_{L}$$ (20)

$$\varepsilon_{f}^{AS}=sgn(\varepsilon )\frac{\sigma_{S}^{AS}}{E}+sgn(\varepsilon )\varepsilon_{L}$$ (21)

$$\varepsilon_{S}^{SA}=sgn(\varepsilon )\frac{\sigma_{S}^{SA}}{E}+sgn(\varepsilon )\nu_{S,n}\varepsilon_{L}$$ (22)

$$\varepsilon_{f}^{SA}=sgn(\varepsilon )\frac{\sigma_{f}^{SA}}{E}$$ (23)

**Figure 6.**
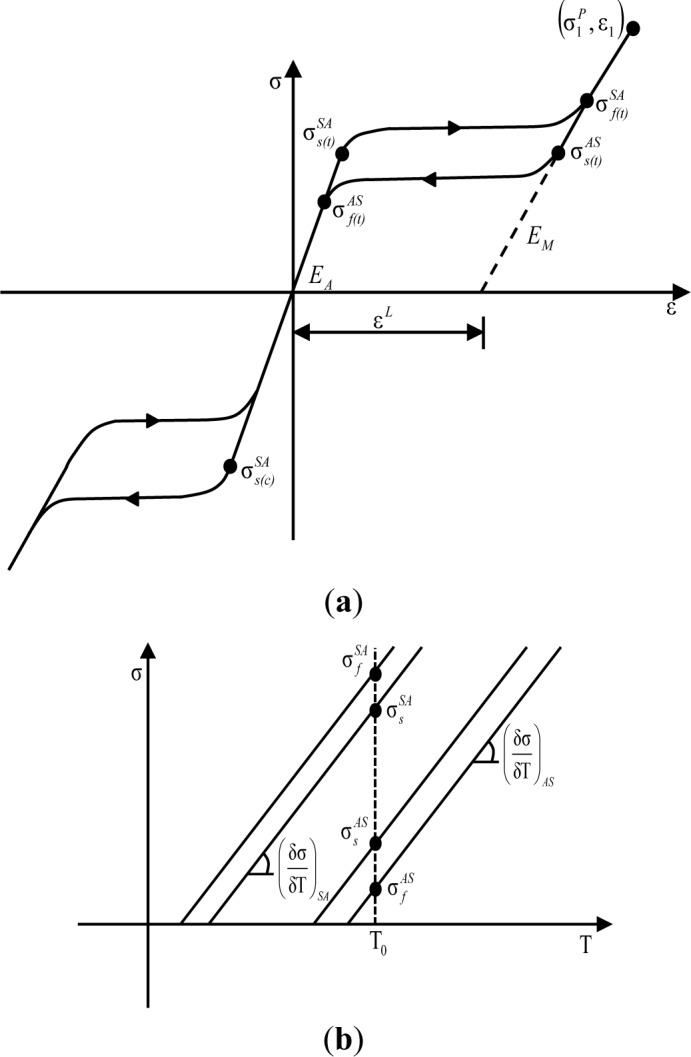
Required parameters used to define the behavior of superelastic SMA materials on the user material (UMAT) subroutine. (**a**) UMAT for simulating the superelastic of behavior of SMA materials under axial loading and (**b**) temperature-dependent phase transformation.

**Figure 7.**
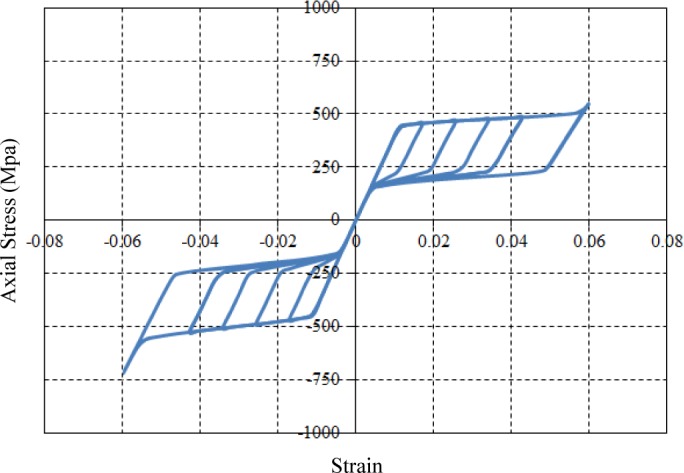
Simulated stress-strain curve for the superelastic SMA material (engineering measurement).

The material data required as input values to the UMAT subroutine are obtained from the observation of uniaxial tests with respect to loading, unloading and reloading under constant temperature. The required parameters used to define the behavior of superelastic SMA materials on the UMAT subroutine are illustrated in Figure 6. The general plasticity was applied to the UMAT algorithm, so that material data in the uniaxial curve should be available at the 3D state during FE analyses. The UMAT code was built in the ABAQUS program associated with a FORTRAN computer language with a view toward numerically simulating the behavior of superelastic SMAs. The simulated stress and strain curve for the superelastic SMA material is shown in Figure 7. In this study, the required material parameters used for simulation—*i.e.*, elastic modulus (40 GPa), Poisson’s ratio (0.33), martensite start stress (440 MPa), martensite finish stress (540 MPa), austenite start stress (250 MPa), austenite finish stress (140 MPa), transformation strain (0.042), temperature (22 °C), and so on—were straightforwardly obtained from uniaxial pull-out tests carried out by DesRoches *et al.* [19].

## 5. Analysis Result and Verification

**Figure 8.**
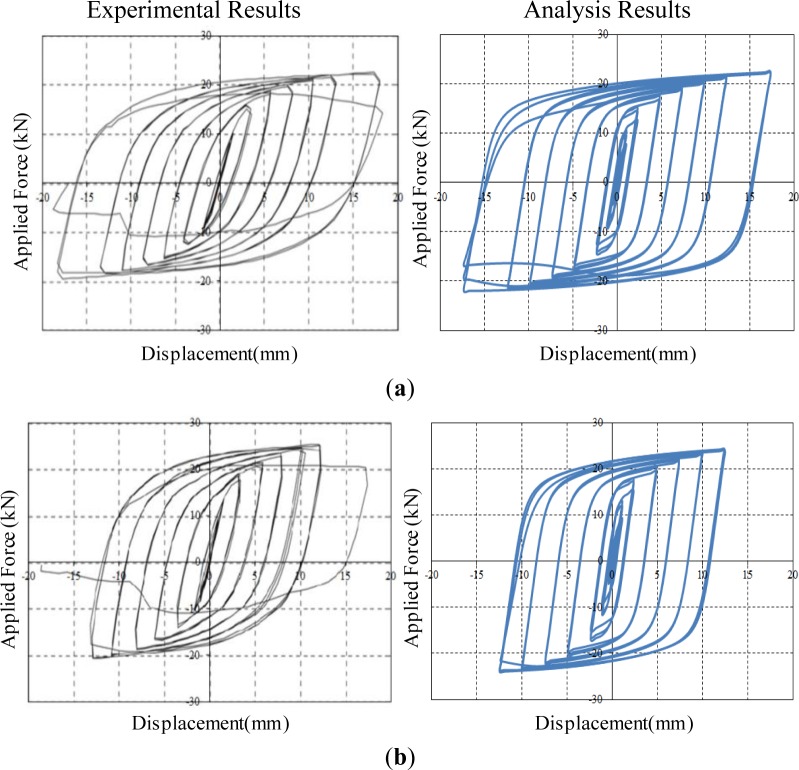

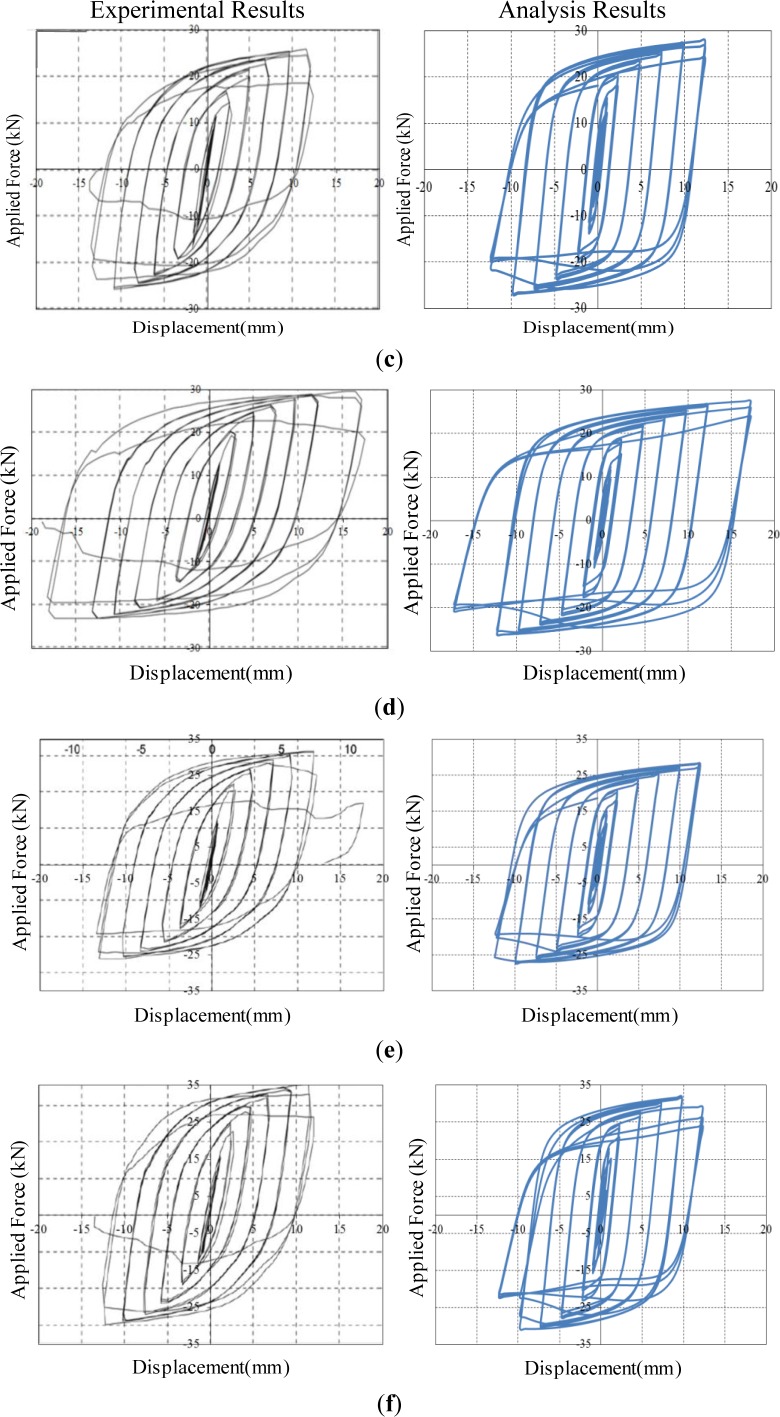
Applied force *vs.* displacement curves for conventional steel slit damper models. (**a**) SL 1 model; (**b**) SL 2 model; (**c**) SL 3 model; (**d**) SL 4 model; (**e**) SL 5 model and (**f**) SL 6 model.

The FE analyses, where both refined solid elements and material nonlinearities are taken into consideration, are able to accurately predict the behavior of slit damper devices subjected to cyclic loading. Figure 8 shows applied force *vs.* corresponding displacement hysteresis curves for steel slit damper models. The detail about force (*P*) and displacement (δ) measurement is presented in Figure 4b. According to individual specimens, analysis results are compared with experimental results in an effort to verify the adequacy of FE modeling under the same displacement loading history. Three cycles were conducted at each amplitude. The experimental tests were carried out until specimens completely failed by fracture.

All specimens for steel slit damper devices have yielded under small displacement loading owing to the inherent characteristics of the base steel materials, thereby dissipating a huge amount of energy. In addition, they exhibit stable hysteretic behavior, including gradual transition and the Bauschinger effect. The SL1 specimen with the smallest *b/l*_0_ ratio withstands the lowest shear force, while the SL6 specimen with the highest *b/l*_0_ ratio sustains the largest shear force. The SL4 specimen with a relatively long *l*_0_ length exhibits excellent ductility, meaning that it has the ultimate displacement of approximately 17.5 mm prior to strength degradation. For the experimental results, strength degradation begins to appear when facture gradually occurs at the ends of the strips, due to stress concentration. The FE models consisting of compatibility-based solid elements with the continuous displacement fields do not include the ability to track the propagation of crack and fracture. For this reason, strength degradation, also observed from the FE analysis results, forms due to geometric nonlinearity rather than fracture after large displacement is imposed on the FE models. Before that occurs, the FE models show symmetric-shaped loops with stable energy dissipation. The parametric ratio of *b/l*_0_ has an influence on the capacity of the FE models as regards strength and ductility, as well. Overall, both resulting curves compared to each other are in good agreement with respect to the initial slope, loading envelope, unloading slope, reloading slope, ultimate strength, permanent deformation and even pinching points for the Bauschinger effect. Further, this good fit between experiment and simulation suggests that the FE models are adequate for predicting the behavior of slit damper devices that are cyclically loaded. Not only the effect of design parameters, but also that of the base materials used, will be investigated through the observation of the FE analysis results.

**Figure 9.**
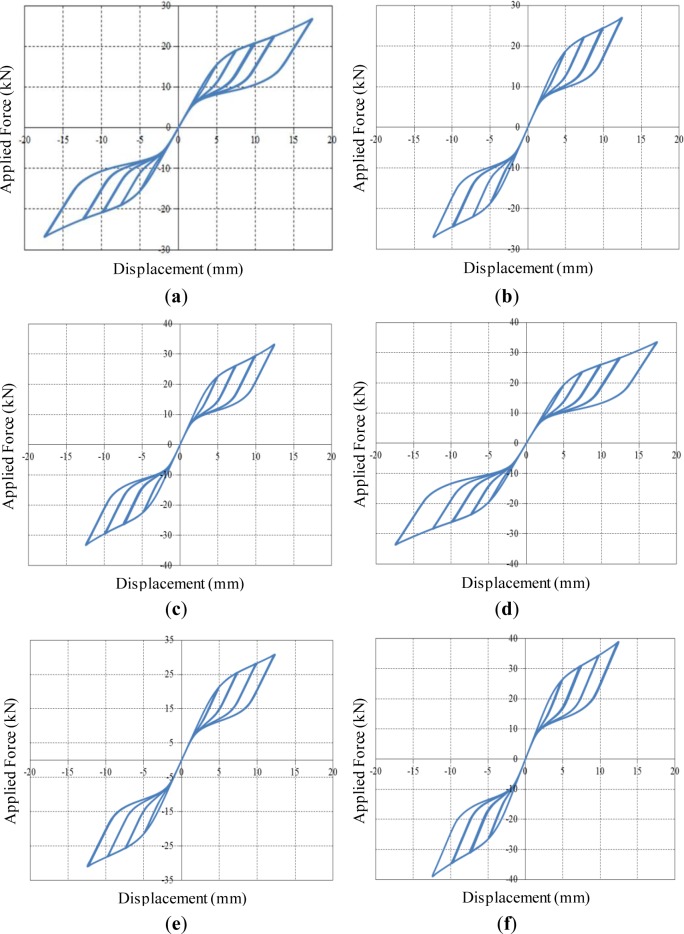
Applied force *vs.* displacement curves for superelastic SMA slit damper models. (**a**) SL-1 SMA model; (**b**) SL-2 SMA model; (**c**) SL-3 SMA model; (**d**) SL-4 SMA model; (**e**) SL-5 SMA model; (**f**) SL-6 SMA model.

Figure 9 shows applied force *vs.* corresponding displacement hysteresis curves for superelastic SMA slit damper models. It is interesting to note that all of the superelastic SMA specimens behave in a similar pattern. They show a unique behavior characterized by a flag-shape hysteresis loop under cyclic loading. As we expected, excellent recentering responses indicating nearly zero permanent deformation upon unloading are observed in the simulating curves. Owing to the restoration of superelastic SMAs, strength degradation does not take place regardless of the geometric nonlinearity. Besides, the superelastic SMA slit damper devices display higher post-yield strength and more flexible stiffness than the steel slit damper devices. It can be clearly shown that the mechanical properties for base materials have a significant influence on the behavior of slit damper devices. The superelastic SMA slit damper devices possess superior performance in terms of flexible initial slope, post-yield strength and recentering behavior compared to the conventional steel slit damper devices. Similar to the steel slit damper devices, superelastic SMA slit damper devices with the relatively higher *b/l*_0_ ratio (*i.e.*, SL6-SMA specimen) can sustain larger shear forces.

The test results are summarized in Tables 2 and 4. The subscripts of “exp” and “ana” in the table denote experiment and analysis, respectively. The yield strength obtained from the experimental results and analysis results (*P*_y,exp_ and *P*_y,ana_) can be determined by finding the intersection of the force-displacement curve with a secant line parallel to the initial slope of the curve (*K*_exp_ and *K*_ana_). The values of yield strength (*P*_y_) calculated by Equation 2 are also tabulated for comparison to the measured properties. The coefficient, *c*, is defined as the ratio of measured stiffness to theoretical stiffness for the fixed-end beam (see Equation 3), as follows:

$$C=\frac{K_{\text{exp}}}{K}\;\text{or}\;\frac{K_{\text{ana}}}{K}$$ (24)

Regardless of the loading direction, both positive (*P*_max_, upward) and negative (*P*_min_, downward) peak strengths are simultaneously tabulated in the analysis results for superelastic SMA slit damper devices, because their simulated hysteresis loops exhibit a perfectly symmetric shape, due to the absence of strength degradation (see also Figure 9). The ductility ratio is defined as the maximum displacement divided by yield displacement, such that *u =* δ_y,exp_*/*δ_max_.

As summarized in the table, the resulting value that is theoretically calculated is close to the finding obtained from experimental tests or FE analyses. Therefore, stiffness coefficients (*c*), as well as normalized yield strength ratios (*P*_y_*/P*_y,exp_ or *P*_y_*/P*_y,ana_) have a value of approximately 1.0. The value of maximum peak strength is on average 2.0 times larger than that of yield strength, because of the material’s strain hardening. For steel slit damper specimens, the yield displacements obtained from experimental tests (δ_y,exp_) are identical to those from FE analyses (δ_y,ana_). Moreover, experimental results for maximum positive strength (*P*_max_) are in good agreement with analytical results for post-yield strength under approximately identical target displacement (+*P*_ana_(+δ_ana_)). The dissipated energy capacity according to individual specimens can be also treated in the same manner. These findings indicate that FE models presented herein are adequate for predicting the behavior of slit damper devices. The specimens fabricated with superelastic SMA material show more flexible stiffness and larger post-yield strength than those fabricated with conventional steel material (e.g., *K*_ana_ = 3.55 kN/mm and *P*_ana_ = 26.80 kN for SL1-SMA specimen *vs. K*_ana_ = 23.77 kN/mm and *P*_ana_ = 22.30 kN for the SL1-SMA specimen), meaning that important characteristics for the behavior of slit damper devices are deeply affected by the properties of the material used. More investigation on the field contours and history outputs will be conducted in the next section.

## 6. Comparison and Observation

**Figure 10.**
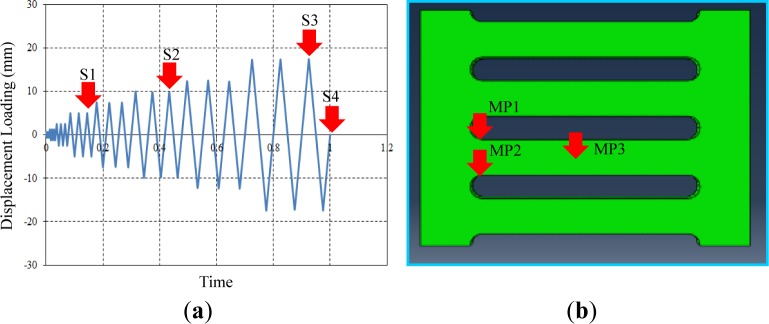
Target displacement for (**a**) field contour observation and (**b**) measurement points. S, stress field contour; MP (Measurement Points).

The test setup for collecting analysis data was developed on the basis of monitoring conditions. Individual target displacements for the field contour observation and measurement points (MPs) for monitoring stress-strain curves are described in Figure 10a,b, respectively. Four target displacements (e.g., S1 = 5 mm, S2 = 10 mm, S3 = 17.5 mm and S4 = 0 mm) were chosen during the cyclic tests performed with displacement loading history. Three set points used for independently measuring uniaxial stress and strain (e.g., MP1, MP2 and MP3) were installed in the FE model, as marked in Figure 10b. The measured data are collected using the history output function provided in the program [13]. Contrary to MP1, which was installed at the middle of the strip, both MP1 and MP2 can detect plastic hinges that generally form at the end of the strip. In particular, the stress-strain curves measured from these set points confirm the validity of the bending mechanism, as elucidated in Figure 4.

**Figure 11.**
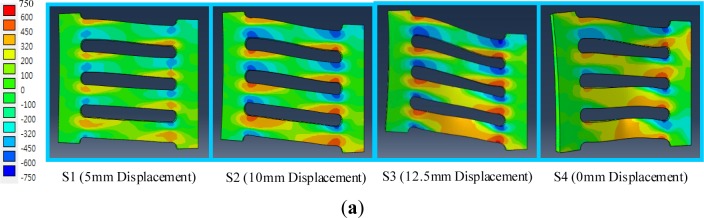

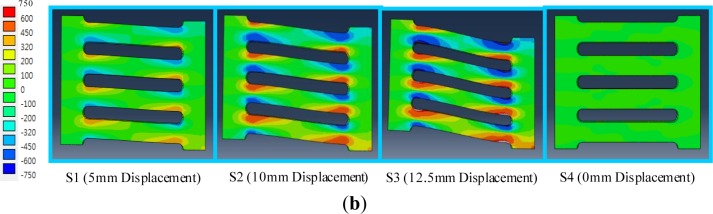
Axial stress components (S11) distributed over the slit damper according to individual displacement loading steps (unit: megapascals): (**a**) SL 1 model; (**b**) SL 1-SMA model.

**Figure 12.**
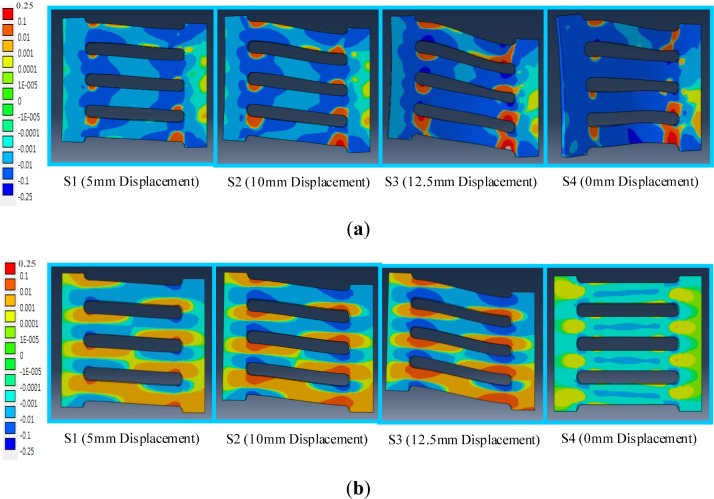
Axial strain components (LE11, logarithmic axial strain field contour) distributed over the slit damper according to the individual displacement loading steps. (**a**) SL 1 model; (**b**) SL 1-SMA model.

The axial stress field contours (S11) distributed over the slit damper according to individual displacement loading steps are shown in Figure 11. The logarithmic axial strain field contours (LE11) are also presented in Figure 12. The SL1 and SL1-SMA specimens are selected for this investigation. The deformed configurations that are particularly necessary to confirm permanent deformation at the final loading step are also found in the figures with a unit deformed scale factor. The colored graph legends are plotted to easily distinguish the magnitude of axial stress and strain contours. The slit damper areas displayed with orange- (for tension) and light blue-colored (for compression) contours have already reached the onset of plastic yielding. Once the amplitude of the loading history exceeds the limit of the yield displacement (δ*_y_*), plastic yielding starts to occur on the strip. For this reason, stress field contours greater than the level of plastic yielding are observed under the first loading step (S1). These axial stress field contours demonstrate that tension and compression yielding are concentrated around both ends of the strips. As the displacement loading increases, plastic stress field areas spread toward the middle of the strips. The red and blue-colored contours, indicating that the base materials are reaching their ultimate tension and compression stress, are found at both ends of the strips when both specimens are compared to each other (SL1 and SL1-SMA specimen) and subjected to the third loading step (S3). In the last loading step (S4), the SL1-SMA specimen completely recovers the original shape without any residual stress distributed over the strips. On the other hand, the SL1 specimen obviously displays out-of-plane deformation that confirms the evidence of instable failure, as well as a considerable amount of residual stress. It may thus be concluded that superelastic SMAs make a good contribution toward decreasing both permanent deformation and residual stress without additional treatment for repair in the case of their utilization in a slit damper device.

As shown in Figure 12, the corresponding logarithm axial strain field contours match the axial stress field contours very well in that they can capture similar yielded regions and plasticity patterns under the same loading step. The base materials that are under plastic yielding are shown with red- (for tension) and blue-colored (for compression) contours. The SL1-SMA specimen has nearly-zero residual strain in the final loading step (S4), while the SL1 specimen shows a lot of residual strain generated due to out-of-plane deformation.

**Figure 13.**
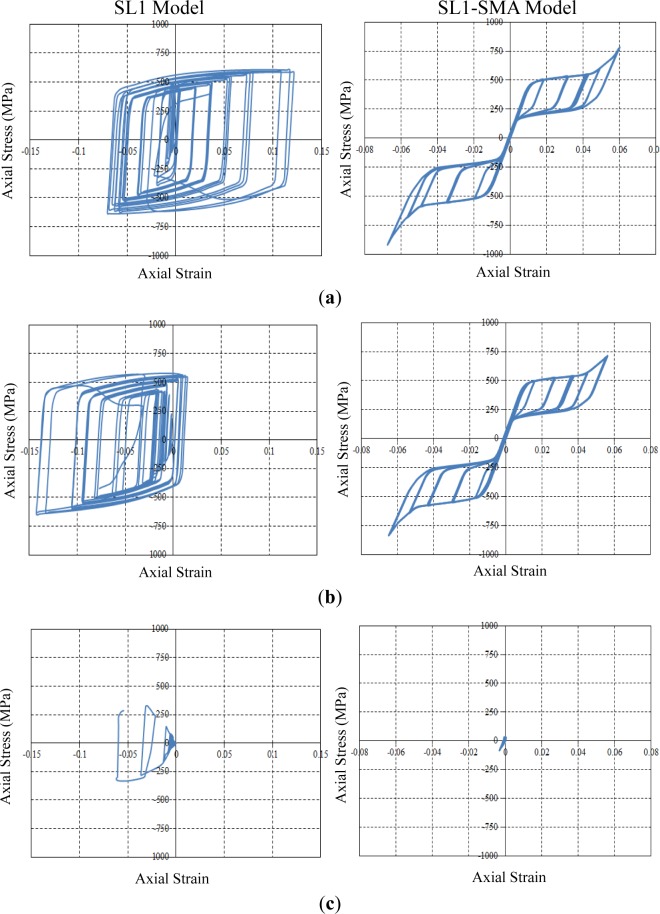
True axial stress and strain curves at the measurement points. (**a**) MP 1 measurement point; (**b**) MP 2 measurement point; (**c**) MP 3 measurement point.

Figure 13 shows the true axial stress and strain curves obtained from the measurement points. For the SL1 specimen, the axial stress and strain curve measured from the MP1 point shifts its center to the right-hand side of the graph. Thus, the MP1 measurement point undergoes tension stress during most of the loading cycles. On the other hand, the MP2 measurement point is under compression stress, as shown in Figure 11a. Both curves exceed the limit of plastic yielding (316.5 MPa). The stress and strain curve measured from the middle of the strip (MP3 point) is under elastic conditions during all loading cycles. However, residual stress taken as 260 MPa exists at this measurement point. For the SL1-SMA specimen, both axial stress and strain curves measured from the MP1 and MP2 points are the symmetric flag-shape hysteresis loops similar to the inherent material behavior. Although both measurement points undergo a considerable amount of plastic deformation upon loading, this specimen can return to the original state upon unloading, owing to its recentering capability. Finally, the MP3 point is also under elastic conditions during all loading cycles. Contrary to the SL1 specimen, the SL1-SMA specimen shows almost zero residual stress, as shown in Figure 13c. It can be thus shown that the UMAT subroutine implemented herein is able to accurately predict mechanical stress, as well as the entire behavior for structures made of superelastic SMA materials.

## 7. Conclusions

Superelastic SMAs as innovative smart materials have been widely applied to passive vibration control devices, because they possess unique and ideal properties, such as a self-healing capacity, attributed to the superelastic effect, supplemental damping guaranteed by the flag-shape hysteresis and outstanding metal fatigue. This paper describes new superelastic SMA slit damper devices with a recentering capability and energy dissipation. For the purposes of simulating the stress-strain curves of superelastic SMA materials, the UMAT subroutine, which can be implemented in the ABAQUS program, is also treated in this study. The behaviors of individual slit damper devices are reproduced by FE analyses. The FE models are calibrated to the established test data aiming for reliable prediction. After obtaining FE analysis results, the slit damper devices fabricated with superelastic SMAs are compared to those made by steel materials with respect to initial flexibility, permanent deformation, strength capacity and residual stress in order to prove that superelastic SMA slit damper devices have superior performance. The FE analysis results demonstrate that conventional steel slit damper devices are susceptible to permanent displacement, residual stress and instability resulting from out-of-plane deformation. However, the proposed SMA slit damper devices overcome these problems. These FE analysis results are promising for the practical application of superelastic SMA slit damper devices, which feature excellent recentering, moderate energy dissipation, nearly zero residual stress and relatively larger post-yield strength. Therefore, outstanding performance, such as the self-centering and vibration control of the device, can be expected in the case of utilizing such smart materials for aseismic design.

## Figures and Tables

**Table 1.** Geometric design parameters for individual SL (Slit Damper) models (Unit: millimeters). SMA, superelastic shape memory alloy.

Model ID	Dimensions			*b*/*l*_0_
	*t*	*b*	*l*_0_	
SL1 (SL1-SMA)	8.0	14.9	97.0	0.155
SL2 (SL2-SMA)	–	15.0	87.1	0.172
SL3 (SL3-SMA)	–	15.1	77.0	0.195
SL4 (SL4-SMA)	–	16.9	99.2	0.172
SL5 (SL5-SMA)	–	16.8	88.3	0.191
SL6 (SL6-SMA)	–	16.5	79.0	0.215

**Table 2.** Summary of the experimental test results (units: kilonewtons and millimeters).

Specimen	*K*_exp_	*c*	*P*_y_	*P*_y,exp_	*P*_y_/*P*_y,exp_	*P*_max_	*P*_min_	δ_y,exp_	δ_max_	μ
SL 1	23.49	0.98	11.59	11.51	1.01	22.61	−19.37	0.49	17.32	35.42
SL 2	33.56	1.00	13.08	13.09	1.00	25.54	−20.59	0.39	12.05	30.86
SL 3	50.07	1.01	15.00	15.02	1.00	25.81	−25.98	0.30	11.66	38.49
SL 4	32.49	1.00	14.58	14.62	1.00	29.61	−23.28	0.45	16.47	36.69
SL 5	44.75	0.99	16.19	16.11	1.00	31.26	−26.40	0.36	11.92	32.83
SL 6	60.24	1.00	17.45	17.47	1.00	35.68	−29.79	0.29	11.44	39.19

**Table 3.** Summary of the analysis results for steel slit damper models (units: kilonewtons and millimeters).

Specimen	*K*_ana_	*c*	*P*_y_	*P*_y,ana_	*P*_y_/*P*_y,ana_	δ_y,ana_	*+P*_ana_ (+δ_ana_)
SL 1	23.77	0.99	11.59	11.41	1.02	0.48	+22.30 (+17.5)
SL 2	34.00	1.01	13.08	12.92	1.01	0.38	+24.21 (+12.5)
SL 3	52.48	1.06	15.00	15.22	0.99	0.29	+27.92 (+12.5)
SL 4	32.49	1.00	14.58	14.62	1.00	0.45	+27.82 (+17.5)
SL 5	44.08	0.97	16.19	16.31	0.99	0.37	+28.82 (+12.5)
SL 6	60.52	1.01	17.45	17.55	0.99	0.29	+31.89 (+10.0)

**Table 4.** Summary of the analysis results for SMA slit damper models (units: kilonewtons and millimeters).

Specimen	*K*_ana_	*c*	*P*_y_	*P*_y,ana_	*P*_y_/*P*_y,ana_	δ_y,ana_	*±P*_ana_ (*±*δ_ana_)
SL 1-SMA	3.55	1.02	16.11	15.80	1.02	4.45	*±*26.80 (*±*17.5)
SL 2-SMA	5.05	1.03	18.19	18.23	1.00	3.61	*±*27.02 (*±*12.5)
SL 3-SMA	7.28	1.00	20.85	21.90	0.95	3.01	*±*33.29 (*±*12.5)
SL 4-SMA	4.34	0.91	20.27	21.80	0.93	5.12	*±*33.69 (*±*17.5)
SL 5-SMA	6.25	0.95	22.50	22.81	0.99	3.65	*±*31.05 (*±*12.5)
SL 6-SMA	8.46	0.97	24.26	24.12	1.01	2.85	*±*38.95 (*±*12.5)
